# The E-domain region of mechano-growth factor inhibits cellular apoptosis and preserves cardiac function during myocardial infarction

**DOI:** 10.1007/s11010-013-1689-4

**Published:** 2013-05-28

**Authors:** Evangelos Mavrommatis, Krystyna M. Shioura, Tamara Los, Paul H. Goldspink

**Affiliations:** 1 Center for Cardiovascular Research, University of Illinois at Chicago, Chicago, IL USA; 2 Department of Physiology and Center for Cardiovascular Research, Medical College of Wisconsin, 8701 Watertown Plank Road, P.O. Box 26509, Milwaukee, WI 53226 USA

**Keywords:** IGF-1 isoforms, E-domain, Myocardial infarction, Cardiac function, Cell death

## Abstract

Insulin-like growth factor-1 (IGF-1) isoforms are expressed via alternative splicing. Expression of the minor isoform IGF-1Eb [also known as mechano-growth factor (MGF)] is responsive to cell stress. Since IGF-1 isoforms differ in their E-domain regions, we are interested in determining the biological function of the MGF E-domain. To do so, a synthetic peptide analog was used to gain mechanistic insight into the actions of the E-domain. Treatment of H9c2 cells indicated a rapid cellular uptake mechanism that did not involve IGF-1 receptor activation but resulted in a nuclear localization. Peptide treatment inhibited the intrinsic apoptotic pathway in H9c2 cells subjected to cell stress with sorbitol by preventing the collapse of the mitochondrial membrane potential and inhibition of caspase-3 activation. Therefore, we administered the peptide at the time of myocardial infarction (MI) in mice. At 2 weeks post-MI cardiac function, gene expression and cell death were assayed. A significant decline in both systolic and diastolic function was evident in untreated mice based on PV loop analysis. Delivery of the E-peptide ameliorated the decline in function and resulted in significant preservation of cardiac contractility. Associated with these changes were an inhibition of pathologic hypertrophy and significantly fewer apoptotic nuclei in the viable myocardium of E-peptide-treated mice post-MI. We conclude that administration of the MGF E-domain peptide may provide a means of modulating local tissue IGF-1 autocrine/paracrine actions to preserve cardiac function, prevent cell death, and pathologic remodeling in the heart.

**Electronic supplementary material:**

The online version of this article (doi:10.1007/s11010-013-1689-4) contains supplementary material, which is available to authorized users.

## Introduction

Ischemia that leads to myocardial infarction (MI) causes cell death and necrosis in the ischemic region, an irreversible loss of muscle tissue and corresponding function. In addition, cardiac myocytes in the viable nonischemic regions undergo programmed cell death known as apoptosis [1]. The heart compensates for this loss of myocardium by undergoing hypertrophic growth to maintain cardiac output. During this remodeling phase, the necrotic and apoptotic regions are replaced with fibrotic tissue which alters ventricular compliance. This further exacerbates the loss of contractility leading to decompensation and heart failure [2].

During these events, local neurohormonal peptides and growth factors such as IGF-1 are produced by the myocardium to mitigate the damage. Insulin-like growth factor 1 (IGF-1) has been extensively shown to inhibit apoptosis in many cell types primarily by activating the IGF-1R/PI3K/Akt pathway [3, 4]. Consistent with this notion are data showing increased IGF-1 mRNA and protein expression in the border zone surrounding a MI in sheep and rats [5, 6]. Myocytes isolated from various regions (border zone and remote regions) show increased expression of IGF-1 supporting the view that IGF-1 expression occurs within the cardiac myocytes [7]. A biphasic response has been reported for IGF-1 mRNA expression in the border zone, peaking initially at day 1, declining and slowly rising by day 7 [5]. However, these studies did not differentiate between the IGF-1 isoforms present, because the hybridization probes or antibodies used only recognize the conserved B, C, A, and D domains of the mature IGF-1 peptide (see Supplemental Data Fig. 1).

IGF-1 is expressed as a prepropeptide growth factor in numerous tissues as different isoforms, but yet isoform regulation and function are poorly understood. Alternate splicing of the transcript gives rises to different classes of transcript based on 5′-end splicing, with different E-domain regions based on 3′-end splicing. With nomenclature based on the different E-domains present in the propeptide form, IGF-1Ea is the predominant isoform [8, 9]. In skeletal muscle, alternate splicing leads to expression of minor isoforms with different E-domain regions in response to growth-promoting stimuli and following injury [10–13]. In the rat heart, temporal regulation of the IGF-1 isoforms has been recently reported following MI, but analysis of the various isoform classes and cardiac myocyte expression was not undertaken. The E-domains of all the isoforms contain a conserved region (first 16 amino acids) derived from exon 4, but diverge thereafter depending on the combination of exons 4–5–6 translated. In rodents, a 52 bp insert from part of exon 5 causes an open reading frame shift and gives rise to the splice-variant IGF-1Eb. IGF-1Eb has also been termed as mechano-growth factor (MGF), due to its upregulation in response to mechanical perturbation [14], and corresponds to the IGF-Ec isoform in humans.

The function and significance of the E-domain regions has become the focus of understanding differential IGF-1 isoform function. Over-expression of full-length cDNAs encoding IGF-1Ea, IGF-Eb (MGF) prepropeptides compared with the mature IGF-1 peptide (exons 3 and 4) results in distinct patterns of gene expression suggesting that the E-domains modulate IGF-1-mediated transcription [15]. Application of a synthetic peptide corresponding to the unique 24 amino acid region of the MGF E-domain has been shown to have distinct biological activity in C_2_C_12_ cells and skeletal muscle precursor cells [16, 17]. In rat, H9c2 myocardial-like cells, the E-domain of MGF exerts autonomous IGF-1 receptor-independent actions [18]. In vivo, intracoronary delivery of the MGF E-domain peptide (E-pep) provides a cardioprotective effect independently and synergistically with mature IGF-1 in an ovine model of MI [19]. A reduction in infarct area and an improvement in function 5-days post-MI was associated with a reduction in caspase-3 activation in the peri-infarct region of MGF E-domain-treated sheep. In addition, systemic delivery of the MGF E-pep has also been shown to produce a neuroprotective effect by preventing apoptosis in a model of transient cerebral ischemia [20]. These data indicate that the E-domain region of MGF may play a role in preventing apoptosis and may provide insight to the function of the MGF isoform.

Consequently, the aim of this study was to examine subclass temporal expression of the IGF-1 isoforms in the heart and cardiac myocytes following MI. In addition, examine E-pep uptake, intracellular location and whether it exerts anti-apoptotic actions in vitro. Finally, we wished assess whether administration could reduce the severity of MI, adverse remodeling and functional decline in mice in vivo. Our data show that the MGF E-pep inhibits the intrinsic apoptotic pathway through mechanisms aimed at preventing the collapse of the mitochondrial resting membrane potential and downstream activation of caspase-3 in H9c2 myocardial-like cells in vitro. Administration of a synthetic peptide at the time of MI prevents cell death in the viable myocardium, preserves cardiac function, and prevents pathologic cardiac hypertrophy. These data indicate that MGF is a physiologically relevant isoform of IGF-1 expressed at the time of injury in cardiac myocytes and suggest that the E-domain region plays a role in mediating the anti-apoptotic effects associated with IGF-1.

## Materials and methods

The experiments were approved by the Institutional Animal Care and Use Committee in accordance with the NIH guidelines.

### E-domain peptides

A 24 amino acid peptide (YQPPSTNKNTKSQRRKGSTFEEHK) corresponding to the unique C-terminal E-domain region of the human MGF isoform (IGF-Ec) was synthesized and purified to >90 % by HPLC (Genescript Corp, NJ). The peptide was stabilized by amidating the C-terminus and switching the arginines at positions 14 and 15 to the d-stereoisomer. A scrambled peptide containing the same amino acids in random order was used as a control in vivo. Two additional peptides were also used for the cell-based studies. A biologically inactive peptide created by substituting the serine at position 18 to alanine (S18A) and an N-terminus FITC-conjugated peptide for the cellular uptake analysis.

### H9c2 myoblasts

Rat H9c2 cells (American Type Culture Collection) were maintained as subconfluent monolayers in growth medium (Dulbecco’s modified eagle medium) supplemented with 10 % fetal bovine serum, plus 1 % penicillin, and streptomycin at 37 °C with 5 % CO_2_. 12 h prior to treatment, the media was changed to DMEM plus 0.2 % BSA and treatments initiated with a fresh change of media. Peptides (50 ng/ml) were either used separately or in combination with recombinant IGF-1 (rIGF-1), purchased from Sigma (100 ng/ml).

### Peptide uptake assay

H9c2 cells were seeded on a 96-well Optilux microtest assay plate (BD Falcon) for 24 h. Media was changed to DMEM + 0.2 % BSA containing FITC-conjugated E-pep and incubated for various times at 37 °C. Cells were washed several times in PBS (plus Ca^2+^Mg^2+^), lysed directly in RIPA buffer (in mM: 150 NaCl, 1 % NP-40, 0.5 % deoxycholic acid, 0.1 % SDS, 50 Tris, pH 8.0) with vigorous shaking for 20 min in the dark at RT. The fluorescence signal was measured at 520 nm in a Bioteck MicroPlate Reader (Synergy HT/190077).

### Immunohistochemistry

Cells grown on coverslips were washed twice in PBS and fixed in 10 % formalin for 10 min. Cells were further washed in several changes of PBS before being permeabilized with PBS containing 0.2 % Tween 20 for 20 min. Nonspecific binding was blocked with 3 % BSA, 2 % goat serum in PBS containing 0.1 % Tween 20 for 30 min at RT. Cells were incubated with a monoclonal antibody that recognizes E-domain region (a kind gift from Dr. V.O. Popov, Russian Academy of Sciences, Moscow, Russia) at 1:10,000 overnight at 4 °C. Following, coverslips were washed and then incubated with goat anti-mouse Alexa Fluor 594 (invitrogen) secondary antibody diluted in PBS containing 2 % BSA for 1 h at RT. Coverslips were mounted in Vectoshield mounting medium with DAPI (Vector Labs) and visualized with epifluorescence using an Olympus IX81 inverted microscope equipped with a Hamamatsu Orca cooled CCD digtial camera, 570–620 and 415 nm band pass filters, and 40× objective.

### Mitochondrial membrane potential (ΔΨ_m_)

The ΔΨ_m_ was assessed using ApoAlert Mitochondrial Membrane Sensor Kit (Clontech). H9c2 cells were seeded in a 96-well microtiter-plate and treated with 0.3 M sorbitol in DMEM (containing 0.2 % BSA and 1 % Pen/Strep). At the end of treatment, cells were rinsed with serum-free media and incubated in 5 μg/ml MitoSensor Reagent for 30 min at 37 °C plus 5 % CO_2_. Cells were washed twice with PBS, resuspended in 100 μl PBS, and analyzed in a microplate reader (Bioteck MicroPlate Reader Synergy HT/190077). The ΔΨ_m_ was assayed for each sample using the ratio of 535 nm (FL1) versus 590 nm (FL2) fluorescence. Data are expressed as the ratio of FL1/FL2 fluorescence.

### Caspase activity assay

Cells were treated with 0.3 M sorbitol for 16 h. Caspase 3 activity was assayed using Caspase Colorimetric Substrate/Inhibitor Quantipak (Biomol International, LP) following the manufacturer’s instructions. Cells were incubated in lysis buffer (in mM: 50 HEPES, 0.1 % CHAPS, 1 DTT, 0.1 EDTA, pH 7.4) on ice for 5 min and then centrifuged at 10,000×*g* for 10 min at 4 °C. Supernatants (10 μl) were loaded on a 96-well microtiter-plate containing 80 μl of assay buffer (in mM: 50 HEPES, 100 NaCl, 0.1 % CHAPS, 10 DTT, 1 EDTA, 10 % Glycerol, pH 7.4). The reaction was started by the addition of Ac-DEVD-pNA (10 μl). OD_405nm_ was measured in 5 min intervals for 2 h in a microplate reader (Bioteck MicroPlate Reader Synergy HT/190077). To measure caspase-8 and -9 activities, specific substrates IETD–pNA and LEHD–pNA were used, respectively, to initiate the reaction.

### MI and peptide treatment

Male B6/SJL 3-month old mice were anesthetized with 3 % isoflurane inhaled in a closed chamber and Etomidate (10 mg/kg, i.p.). Mice were intubated and connected to a rodent ventilator and additional anesthesia was regulated by the delivery of 1.5 % isoflurane through a vaporizer with 100 % oxygen. The left anterior descending coronary artery was ligated with 8-0 silk suture 1–2 mm below the left atrium, as previously published [21]. Peptides were administered via micro-osmotic pumps (Alzet, Model 1007D, Durect Corp, CA) implanted subcutaneously, slightly posterior to the scapula under general anesthesia. Peptides were dissolved in 25 % mouse serum in 0.9 % NaCl at concentrations sufficient to allow an infusion rate of 4.5 mg/Kg/day. Untreated control mice and infarcted mice received vehicle via micro-osmotic pump. Pumps were implanted 12 h before coronary artery ligation to ensure appropriate dose, and replaced after 7 days for 2 week treatments.

### Myocyte isolation

While under anesthesia, mice were heparinized (5,000 units/kg), and the heart was quickly removed and placed in a ice-cold nominal Ca^2+^-free control solution (in mM: NaCl 133.5, KCl 4, NaH_2_PO_4_ 1.2, MgSO_4_ 1.2, HEPES 10, and glucose 11). The aorta was cannulated and the heart mounted on a Langendorff apparatus and perfused at constant temperature 37 °C and flow of 3 ml/min for 4 min with Ca^2+^-free control solution containing (in mM: 113 NaCl, 4.7 KCl, 0.6 KH_2_PO_4_, 0.6 Na_2_HPO_4_, 1.2 MgSO_4_·7H_2_O, 12 NaHCO_3_, 10 HEPES, 30 taurine, 0.032 phenol red, 10 butanedione monoxime (BDM), and 5.5 glucose) followed by an identical buffer containing additional enzymes (0.25 mg/ml Blendzyme I (Roche), 0.14 mg/ml trypsin, and 12.5 μM CaCl_2_) for 8 min. Hearts were removed, minced in digestion buffer, then pipetted for 1–2 min until the cells were completely dissociated. The cell suspension was rinsed in stop buffer (control buffer plus 5 % of bovine serum albumin) to inactivate the enzymes.

### Western blotting

Freshly isolated adult cardiac myocytes were lysed in buffer (in mM: 20 Tris–HCl (pH 7.4), 2 EDTA, 10 EGTA, 320 sucrose, 0.3 PMSF, 20 μg/ml leupeptin, and 10 β-mercaptoethanol). Protein quantification was determined by the DC Protein Assay (Bio-Rad). Samples diluted 1:2 with Laemmli sample buffer and heated (68 °C) for 5 min. Proteins were separated on a 10–20 % gradient gel. H9c2 cells were lysed in RIPA buffer, containing Protease Inhibitor Cocktail Set I (Calbiochem) and Phosphatase Inhibitor Cocktail 1 (Sigma-Aldrich), prior to SDS-PAGE on 12 % acrylamide gels before transferring to nitrocellulose. Membranes were incubated in blocking buffer consisting 5 % nonfat dry milk in TBS (in mM: 20 Tris–HCl, 137 NaCl, pH 7.6) for 1 h. Affinity-purified antibodies against phospho-IGF-1 receptor (Tyr^980^ and Tyr^1131^) phospho-Akt (Ser^473^), actin (cell signaling), and the E-domain monoclonal antibody were used at 1:1,000 overnight at 4 °C. After washing membranes in TBS-T, secondary antibody (goat anti-rabbit diluted 1:1,000 in blocking buffer) was added for 1 h at RT. Immunoreactive bands were detected with SuperSignal West Pico Chemiluminescent Substrate (Pierce) and visualized using autoradiographic film.

### Cardiac function

A 1.4 French pressure–conductance catheter (SPR-839, Millar Instruments, Houston, TX, USA) was inserted retrograde into the left ventricle and pressure–volume loops recorded (ARIA Pressure Volume Conductance System, Millar Instruments) in anesthetized mice as described [21, 22].

### Quantitative RT-PCR

Total RNA was extracted from the apex of the heart with TRIzol (Invitrogen), and used in a one-step RT-PCR reaction with the SYBR Green RNA Amplification kit (Roche Molecular Biochemical, IN) in the LightCycler thermocycler (Roche Diagnostics). The reaction conditions for the reverse transcriptase were 55 °C for 15 min, a denaturing step at 95 °C for 30 s, followed by four-step PCR amplification for 40-cycles. The second derivative maximum (log linear phase) for each amplification curve was plotted against a standard curve to calculate the amount of product. Samples from mouse hearts were normalized against GAPDH and H9c2 cells normalized against ribosomal protein L7, as previously described [21, 22]. Primers were designed using GenBank reference sequences and the primer design tool Primer 3 (http://frodo.wi.mit.edu/cgi-bin/primer3/primer3_www.cgi). Primer sequences were run against the BLAST database and used for melting curve analysis prior to PCR amplification to ensure single gene specific product amplification.

**Table:** 

Gene	Forward primer	Reverse primer	Genebank accession number
IGF-1Ea	GCTTGCTCACCTTCACCAGC	AAATGTACTTCCTTCTGAGTCT	NM_001111275
IGF-1Eb (MGF)	GCTTGCTCACCTTCACCAGC	AAATGTACTTCCTTTCCTTCTC	NM_010512
Class I-MGF	ATGGGGAAAATCAGCAGCC	CACCATCATGTCGTCCCACACCTCTT	NM_010512
Class II-MGF	ATGACCGCACCTGCAATAAAG	CACCATCATGTCGTCCCACACCTCTT	NM_001111274
α-Myosin heavy chain	AAGGTGAAGGCCTACAAGCG	TTTCTGCTGGACAGGTTATTCC	M76601
β-Myosin heavy chain	AAGGTGAAGGCCTACAAGCG	TTCTGCTTCCACCTAAAGGGC	M74752
Atrial natriuretic factor	TGGAGGAGAAGATGCCGGTA	CGAAGCAGCTGGATCTTCGTAG	K02781
Matrix metalloproteinase 2	AGCTCCCAGAAAAGATTGACG	GATGAGCTTAGGGAAACCGGG	NM_008610
Glyceraldehyde-3-phosphate dehydrogenase	TATGACAATGAATACGGCT	CTCCTGTTATTATGGGGG	M32599
Rat IGF-1 Eb (MGF)	GCTTGCTCACCTTCACCAGC	AAATGTACTTCCTTTCCTTCTC	NM_001082478
RPL7	GAAGCTCATCTATGAGAAGGC	CAGACGGAGCAGCTGCAGCAC	NM_001100534

### In situ cell death

Hearts were removed, fixed in 10 % buffered formalin and 5 μm sections cut from paraffin-embedded blocks. TUNEL staining was conducted using the In situ Cell Death Detection Kit TMR Red (Roche), according to the manufacture’s instructions with slight modifications. Sections were deparaffinized and incubated with Retrievagen A buffer (BD Pharmingen) at 86 °C for 12 min and then cooled to RT for 20 min. Sections were washed with PBS for 15 min and the enzyme mix applied for 10 min at 37 °C. Slides were washed and mounted with DAPI. Three digital images from the infarct border zone and viable myocardium were taken and analyzed. Digital visualization, analysis, and calculations were performed using Volocity software (Improvision). The numbers of nuclei in each field were highlighted by selecting a pixel in the middle of the nucleus and drawing the region of interest outwards (Drawing Region of Interest Tool) until the maximum threshold was reached. Tolerance was set at 25 % similar to the picked voxel for all objects and objects smaller than 3 μm were excluded from final analysis. Colocalization of signal within the same voxel location in each channel was used to determine the number of total and apoptotic nuclei in the same image field. Data are expressed as percent apoptotic nuclei.

### Infarct quantification

Duplicate 1 mm mid-LV sections were cut and incubated with 1 % triphenyl tetrazolium chloride (TTC) for 20 min at 37 °C. The infarct size was determined by planimetry of the infarct zone (white) and expressed as a percent of the total LV area on digital images at 10× magnification (Advanced SPOT Diagnostic Instruments) [21].

### Statistics

Data are expressed as means ± standard error (SE). The difference in the mean was tested using either Student’s *t* test or ANOVA followed by Newman–Keuls post hoc analysis, where appropriate. Cardiac function data were analyzed using two-way ANOVA to test the influence of two independent variables: infarct and treatment. All pairwise multiple comparisons were performed using the Holm-Sidak method to test for statistical significance *(P* < 0.05).

## Results

### The MGF isoform is expressed in response to cell stress in vitro

**Fig. 1:**
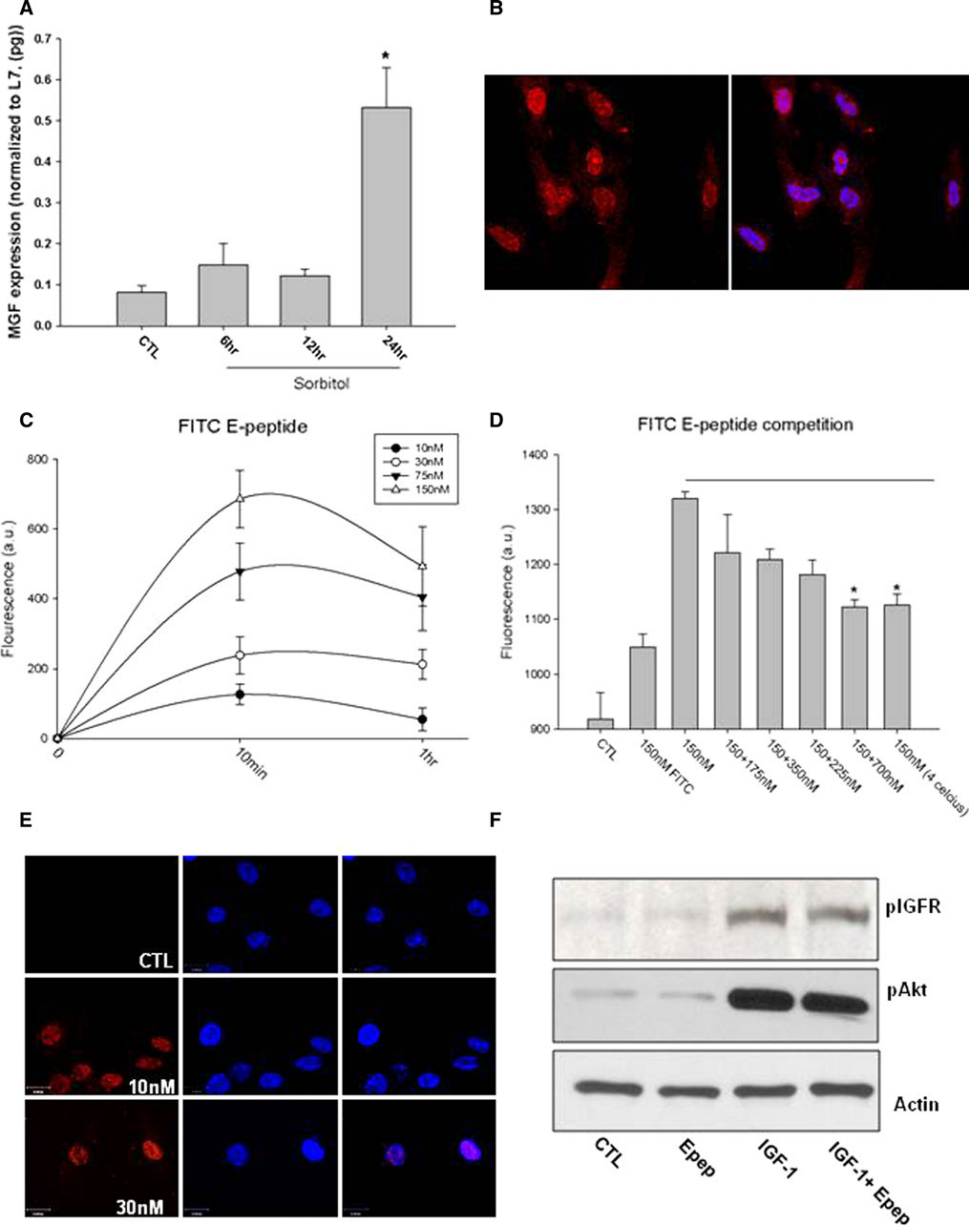
**a** Endogenous MGF isoform mRNA expression following exposure to 0.3 M sorbitol in H9c2 cells (*P < 0.05 vs. control, *n* = 3). **b** Immunostaining with MGF E-domain-specific antibody in H9c2 cells following 24 h sorbitol treatment (*Red* is E-domain specific antibody, *blue* is DAPI). **c** Quantification of FITC-conjugated MGF E-domain peptide uptake in H9c2 cells with various concentrations. **d** Competition assay with excess unlabeled MGF E-domain peptide with 10 min incubation, FITC alone and incubation at 4 °C (*P < 0.05 vs. control, *n* = 5). **e** Immunostaining of cells treated with the E-domain peptide (30 nM) for 15 min with an MGF E-domain-specific antibody and counter stained with DAPI. **f** Immunoblot of H9c2 cell lysates treated with MGF E-domain peptide, IGF-1 or MGF E-domain peptide + IGF-1 for 15 min. Anti-pIGF-1R (Tyr^980^), anti-pAkt (Ser^473^) to examine IGF-1 receptor and pathway activation. Anti-actin shows equal loading among samples. (Color figure online)

Studies have shown that sorbitol treatment induces osmotic shock, oxidative, metabolic stress, mitochondrial calcium overload which all lead to apoptosis in H9c2 rat cardiomyoblasts and cultured cardiac myocytes [23–29]. To ascertain whether the endogenous MGF isoform is expressed in H9c2 cells in response to cell stress, cells were treated with 0.3 M sorbitol. MGF isoform expression significantly increased by 24 h suggesting that the IGF-1 isoform splicing was responsive to cell stress in H9c2 cells. Immunohistochemical analysis using an E-domain-specific antibody revealed a perinuclear and nuclear localization of the MGF E-domain (Fig. 1a, b).

### MGF E-domain is taken up by the cell without IGF-1 receptor activation

Given several biological processes have been reported to be regulated by the 24 a.a. peptide derived from the E-domain region of MGF, we examined cellular uptake using a FITC-labeled E-pep. Treatment resulted in a dose-dependent increase in cellular fluorescence, which peaked at 10 min (Fig. 1c). Competition of FITC-labeled E-pep with increasing concentrations of unlabeled peptide resulted in a significant decrease in fluorescence with a 4-fold concentration of unlabeled E-pep (Fig. 1d). To rule out the possibility of nonspecific electrostatic interaction, cells were treated with FITC alone and at 4 °C. Lowering the temperature to 4 °C solidifies the plasma membrane and prevents endocytosis or receptor internalization. FITC alone produced a low level of fluorescence, whereas incubation at 4 °C significantly decreased the amount of FITC-labeled peptide able to enter the cell (Fig. 1d). Staining with an E-domain-specific antibody revealed nuclear accumulation of the peptide within 15 min, which persisted for up to 24 h (data not shown) (Fig. 1e).

Since MGF is a splice variant of IGF-1, the impact of E-pep treatment on IGF-1R/Akt signaling was examined. Treatment with the E-pep did not activate the IGF-1 receptor as measured by phosphorylations at Tyr^980^ (Fig. 1f) and Tyr^1131^ (data not shown) whereas IGF-1 alone did. Application of the propeptide form (IGF-1 + E-domain) showed no apparent difference in receptor phosphorylation compared with IGF-1 alone. A similar effect was also noted with respect to Akt phosphorylation at serine 473 (Fig. 1f).

### MGF E-domain inhibits the intrinsic apoptotic pathway in vitro

**Fig. 2:**
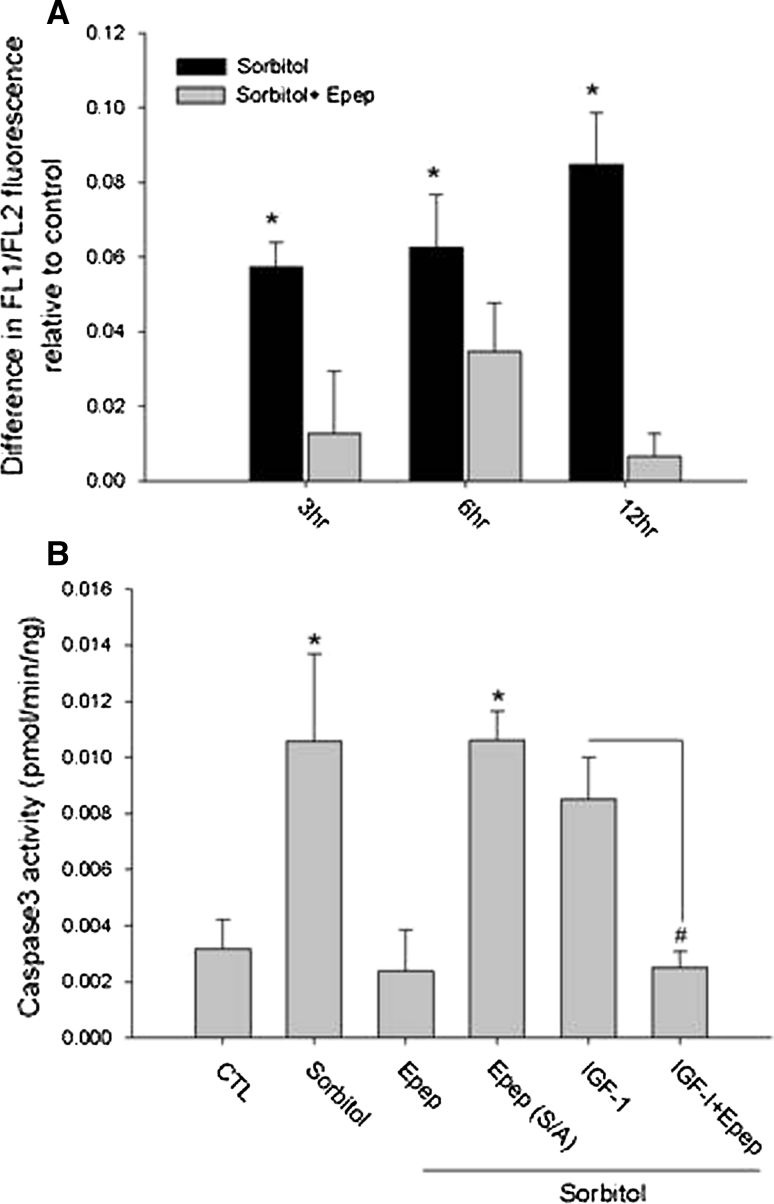
MGF E-peptide exerts anti-apoptotic actions in H9c2 cells. **a** Mitochondrial membrane potential in response to 0.3 M sorbitol and MGF E-domain peptide treatment for various times (*P < 0.05 vs. sorbitol + MGF E-domain). **b** Caspase-3 activity in response to sorbitol treatment (12 h), with MGF E-domain peptide and MGF E-domain peptide containing an amino acid substitution (S/A), (*P < 0.05 vs. control, ^#^ *P* < 0.05 vs. IGF-1, *n* = 5)

Previous data from sheep treated with the E-pep following MI suggested that the E-pep may exert anti-apoptotic actions [19]. H9c2 cells were treated with sorbitol and the mitochondrial membrane potential (ΔΨ_m_) analyzed. A progressive increase in the fluorescence ratio with time of sorbitol treatment indicated a collapse of the ΔΨ_m_ was prevented with E-pep treatment (Fig. 2a). The collapse of the ΔΨ_m_ signals the onset of the apoptotic cascade due to release of cytochrome c into the cytoplasm, which activates caspase-9. Caspase-9 activity was significantly elevated in response to sorbitol but inhibited with E-pep treatment (Supplementary Data Fig. 2a). E-pep treatment of the extrinsic pathway via caspase 8 showed no effect, indicating an inhibition of the intrinsic apoptotic pathway (Supplementary Data Fig. 2b). Since caspase-3 is a terminal enzyme in the apoptosis cascade and receives input from upstream caspases, we assessed its activation as an index of cell fate. Sorbitol-induced caspase-3 activation which was prevented with E-pep treatment. This effect appeared to be more potent than IGF-1 treatment alone and when added in combination with IGF-1 did not provide an additive effect. These data suggest that the E-domain may engage the IGF-1 cell survival pathway with different spatial–temporal dynamics or different survival pathways to prevent caspase-3 activity (Fig. 2b). To define whether the actions of the E-pep were specific, a peptide in which the serine at position 18 was substituted to alanine (S18A) was used. Substitution of the terminal serine rendered the E-pep biologically inactive as indicated by an inability to inhibit caspase-3 activity.

### The MGF isoform is induced immediately following MI

**Fig. 3:**
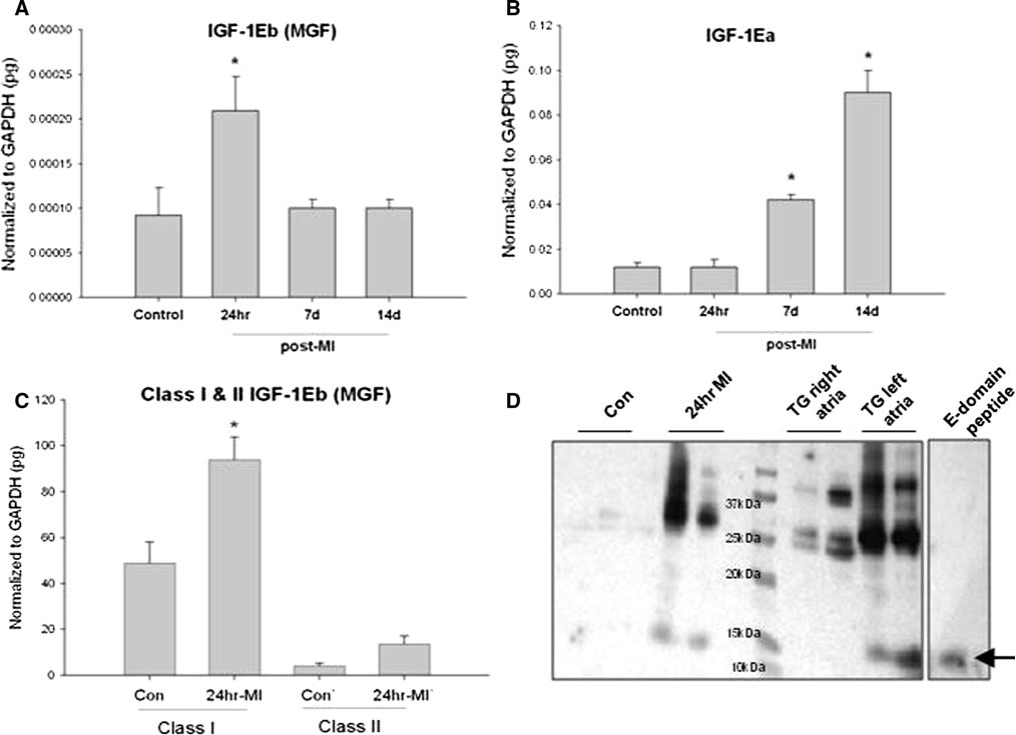
Expression and quantification of IGF-1 isoform expression in the mouse heart following MI. **a**, **b** Quantification of the 3′-splice variants using isoform specific primers at various times post-MI (*P < 0.05, *n* = 4). **c** Analysis of 5′-splice variants of the MGF transcript (rodent IGF-1Eb), with class/isoform specific primers 24 h post-MI (*P < 0.05). **d** Western blot of myocyte protein extracts isolated from control and 24 h post-MI hearts (duplicate samples). Protein extracts from the atria of a PKCε transgenic mouse run as positive controls (*arrow* indicates migration of fragment <15 kDa). The synthetic E-domain peptide was also run under similar conditions and probed with an MGF E-domain-specific antibody

To characterize the expression of the IGF-1 isoforms expressed in the mouse heart following MI, isoform specific primers were used. There was a significant increase in IGF-1Eb (MGF) expression 24 h post-MI, while expression of IGF-1Ea did not change (Fig. 3a, b). Analysis up to 2 week post-MI showed IGF-1Ea was significantly increased 7 and 14 days post-MI, while MGF expression declined to baseline levels (Fig. 3b, c). Finally, analysis of the 5′-splice variants revealed that the class-1 MGF transcript predominates and is significantly increased 24 h post-MI (Fig. 3d).

To determine whether MGF protein expression was increased, the MGF E-domain specific antibody was used to probe isolated myocyte protein extracts (Fig. 3e). In addition, protein from the atria of a previously characterized transgenic mouse model that develops heart failure was used as a biological source for comparison [22]. The left atrial enlargement noted in this model is due to the development of a thrombus but expresses high levels of MGF transcript (data not shown). Multiple bands were identified in both 24 h post-MI myocyte and left atrial protein extracts, likely representing the prepropeptide and propeptide forms of MGF. The presence of a faster migrating band below 15 kDa in protein extracts similar to the synthetic E-pep, suggests that the E-domain region of MGF is cleaved in myocytes following MI.

### MGF E-domain treatment preserves contractility and cardiovascular function post-MI

To examine the salutary actions of E-pep treatment in vivo, we delivered the stabilized peptide to infarcted mice. Pressure–volume loop analysis was performed to assess cardiovascular function in mice treated with the E-pep, or a scrambled peptide (SCR-pep), at 2 week post-MI. Hemodynamic variables measured in all control groups (control, control + SCR-pep, control + E-pep) showed no differences after 2 week treatment. Analysis of MI + SCR-pep showed no differences compared with MI alone, whereas MI + E-pep treatment did show significant differences (Table 1).

**Table 1:** Cardiac function in mice at 2 week post-MI with and without MGF E-peptide treatment

Group parameter	Control	Control + SCR-peptide	Control + E-peptide	2 week MI	2 week MI + SCR-peptide	2 week MI + E-peptide
HR	594 ± 20	546 ± 25	526 ± 24	555 ± 17	565 ± 27	530 ± 16
MAP	80 ± 2.5	71 ± 1.9	74 ± 1.3	61 ± 5*	66 ± 4.0*	76 ± 3.6^**†**^
EDV	46 ± 1.0	47 ± 4.0	50 ± 3.0	66 ± 3.1*	69 ± 3.0*	68 ± 2.4*
ESV	25 ± 0.7	24 ± 0.8	27.5 ± 1.0	53 ± 3.3*	58 ± 2.5*	55 ± 3.5*
*V*_int_	22.4 ± 1.4	21.2 ± 0.8	24 ± 1.3	38.9 ± 4*	49 ± 2.7*	39.6 ± 3.5*
ESP	102 ± 2.2	102 ± 8.8	93 ± 2.0	77 ± 3.1*	79 ± 3.0*	92 ± 3.5^**†**^
EDP	6.8 ± 0.7	7.0 ± 1.0	6.7 ± 0.8	9.7 ± 1.5*	9.7 ± 1.7*	8.2 ± 1.4*
SV	20.8 ± 0.7	22.6 ± 2.7	22.4 ± 1.0	12.2 ± 1.7*	12.9 ± 0.7*	15.1 ± 1.4*
CO	12,138 ± 972	11,879 ± 1,835	11,891 ± 742	6,987 ± 871*	6,850 ± 650*	7,938 ± 933*
SW	1,645 ± 60	1,749 ± 199	1,645 ± 170	645 ± 72*	713 ± 77*	1,236 ± 154*^,**†**^
d*P*/dt_max_	11,321 ± 560	10,053 ± 346	8,957 ± 337	5,692 ± 311*	6,085 ± 409*	10,357 ± 240^**†**^
d*P*/dt_min_	−10,344 ± 617	−8,704 ± 237	−8,632 ± 235	−5,562 ± 546*	−5,988 ± 418*	−8,918 ± 285^**†**^
Tau-G	8.3 ± 0.6	8.7 ± 1.1	7.3 ± 0.3	15.4 ± 3.5*	10.3 ± 0.8*	9.7 ± 0.5*
PAMP	60.6 ± 7.6	55.6 ± 5.3	48 5 ± 6.1	9.6 ± 1.2*	8.6 ± 1.0*	23.0 ± 2.0*
TPR	6.8 ± 0.6	6.3 ± 0.7	6.4 ± 0.5	10.2 ± 1.6*	10.4 ± 1.3*	9.3 ± 1.0*
*E*_a_	4.6 ± 0.23	4.6 ± 0.8	4.5 ± 0.5	6.6 ± 0.6*	7.7 ± 0.7*	7.5 ± 0.6*
*E*_a_/*E*_s_	0.20 ± 0.01	0.23 ± 0.04	0.28 ± 0.04	0.84 ± 0.06*	0.71 ± 0.11*	0.60 ± .0.04*^,**†**^

Pressure–volume loop measurements collected in the closed chest configuration

*HR* Heart rate (beats per minute), *MAP* mean arterial pressure (mmHg), *EDV* end diastolic volume (μl), *ESV* end systolic volume (μl), *V*_*int*_ volume intercept (μl), *ESP* end-systolic pressure (mmHg), *EDP* end-diastolic pressure (mmHg), *SV* stroke volume (μl), *CO* cardiac output (μl/min), *SW* stroke work (mmHg/μl), *dP/dt*_*max*_ maximum first derivative of change in systolic pressure with respect to time (mmHg/s), *dP/dt*_*min*_ maximum first derivative of change in diastolic pressure with respect to time (mmHg/s), Tau-Glantz-time constant of fall in ventricular pressure by Glantz method (ms), *PAMP* preload adjusted maximal power (mWatts/ml^2^), *TPR* total peripheral resistance (mmHg/ml/min), *E*_a_ */E*_s_ vascular-to-ventricular coupling

* *P* < 0.05 versus control; ^**†**^ *P* < 0.05 versus 2 week MI (*n* = 6 per group)

Overall, there was a significant decline in cardiac function in both MI- and MI + SCR-pep-treated mice compared with controls. This was reflected in the increased end systolic and end diastolic volumes, decreased systolic function (ESP, SV, CO, and SW) and increased diastolic dysfunction (EDP, Tau). Treatment with the E-pep resulted in a number of hemodynamic variables being either preserved or attenuated, despite similar signs of early dilation, based on the volume intercept (*V*_int_). The pressures developed (MAP, ESP, and EDP) were either similar to those measured in control mice or attenuated. While other functional parameters declined, particularly with respect to volumes (SV and CO), they did so to a lesser extent than the MI and MI + SCR-pep-treated groups (Table 1). In addition, measurements of load-dependent cardiac contractility or work (stroke work, d*P*/dt_max_, d*P*/dt_min_, PAMP) were statistically improved in MI + E-pep-treated mice compared with MI and MI + SCR-pep-treated mice (Table 1).

**Fig. 4:**
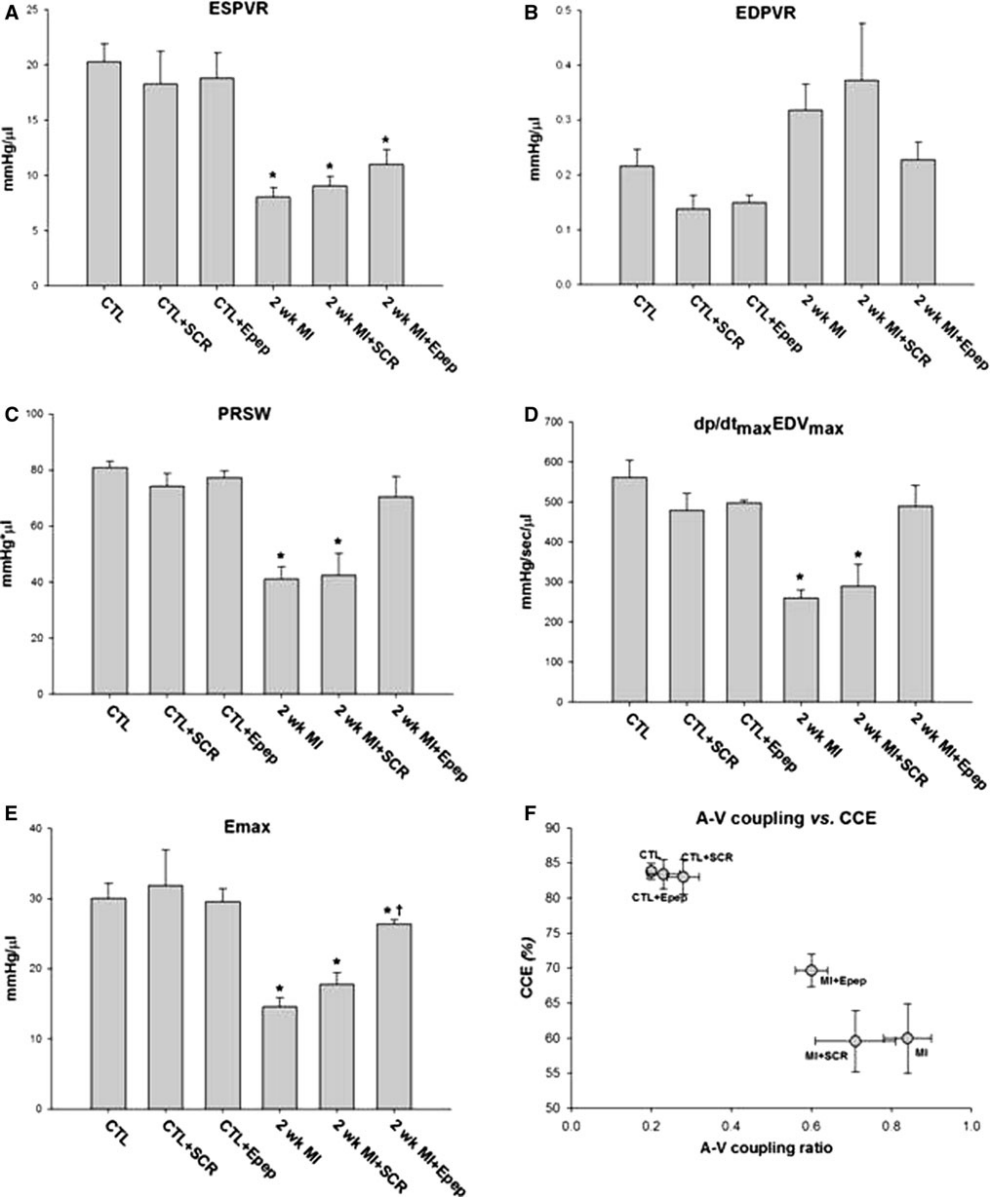
Cardiac contractility based on P–V loop measurements collected during transient occlusion of thoracic vena cava in 2 week post-MI mice with and without MGF E-peptide treatment. **a** ESPVR-end systolic pressure volume relationship. **b** EDPVR-end diastolic pressure volume relationship. **c** PRSW-preload recruitable stroke work. **d** d*P*/dt_max_ versus EDV_max_-maximal d*P*/dt versus maximal end diastolic volume. **e** *E*_max_-time-varying maximal elastance. **f** The relationship between A–V coupling ratio (*E*_a_/*E*_s_) and cardiac contractility efficiency (CCE) in all 2 week post-MI mice (*P < 0.05 vs. control and ^†^ *P* < 0.05 vs. 2 week MI, *n* = 6)

Measures of cardiac contractility obtained during thoracic vena cava occlusion, showed the end-systolic pressure volume relationship (ESPVR) was significantly depressed in MI mice but to a lesser extent in MI + E-pep mice (Fig. 4a). The end-diastolic pressure volume relationship (EDPVR) was elevated in MI and MI + SCR-pep mice but did not differ from control in MI + E-pep mice (Fig. 4b). Preload recruitable stroke work (PRSW), reveals changes in systolic function independent of chamber geometry, was preserved in MI + E-pep-treated mice (Fig. 4c). Likewise, maximal d*P*/dt versus maximal EDV (d*P*/dt_max_ versus EDV_max_) was also preserved in the MI + E-pep-treated mice (Fig. 4d). Time-varying elastance (*E*_max_) indicates the temporal course of the chamber stiffness, decreased significantly in post-MI mice but was attenuated in MI + E-pep-treated mice (Fig. 4e). Finally, assessment of global cardiovascular function expressed as the vascular–ventricular (A-V) coupling ratio (*E*_a_/*E*_s_), revealed significant differences. The *E*_a_/*E*_s_ ratio was significantly increased in MI and MI + SCR-pep mice (Table 1). An increase in the *E*_a_/*E*_s_ ratio suggests that an increase in vascular resistance is occurring along with a decrease in mechanical efficiency of the ventricle. However, the *E*_a_/*E*_s_ ratio was significantly lower in MI + E-pep mice and plotted against cardiac contractile efficiency indicates that an improvement in mechanical efficiency of the ventricle occurred independently of vascular changes (Fig. 4f).

### MGF E-domain treatment prevents cardiac hypertrophy post-MI

**Fig. 5:**
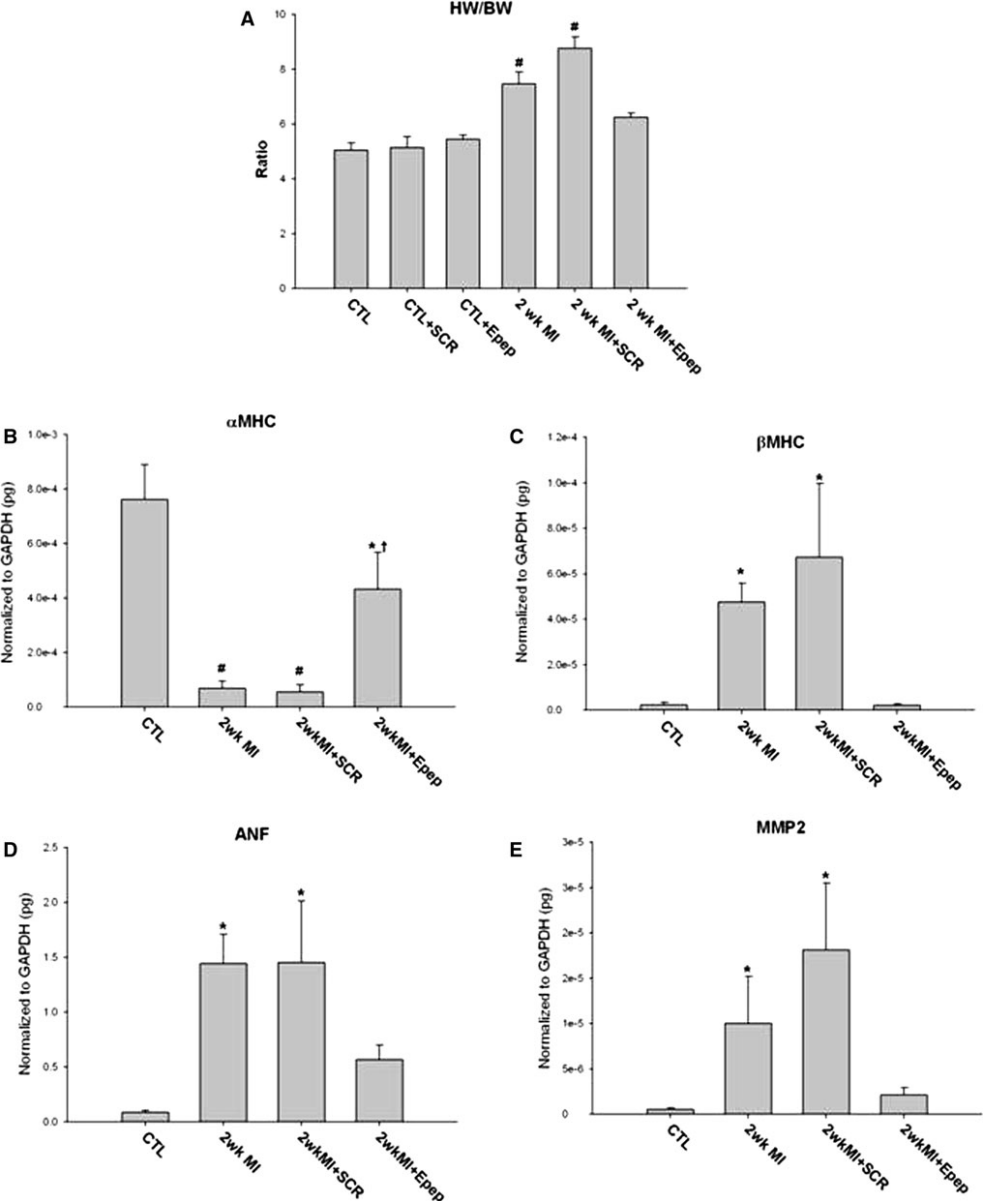
Quantification of cardiac mass and gene expression in 2 week post-MI mice with and without MGF E-peptide treatment. **a** Heart weight to body weight ratios. **b** α-myosin heavy chain isoform expression. **c** β-myosin heavy chain isoform expression. **d** ANF expression. **e** Metalloproteinase expression MMP2 (*P < 0.05, ^#^ *P* < 0.01 vs. control, *n* = 5)

Analysis of heart weight and molecular markers was used to assess the extent of cardiac hypertrophy. A significant increase in the heart weight to body weight ratio (HW/BW) in MI and MI + SCR-pep mice was inhibited in MI + E-pep mice (Fig. 5a). Analysis of myosin heavy chain isoform expression showed preserved α-MHC isoform expression in MI + E-pep-treated hearts compared with MI and MI + SCR-pep-treated hearts. Conversely, there was a reciprocal increase in β-MHC isoform expression in MI and MI + SCR-pep hearts, which was suppressed in MI + E-pep-treated hearts (Fig. 5b, c). A significant increase in ANF expression evident in MI and MI + SCR-pep was prevented in MI + E-pep-treated hearts (Fig. 5d). Analysis of the matrix metalloproteinases implicated in extracellular matrix remodeling post-MI showed that MMP-2 mRNA expression was significantly increased in MI and MI + SCR-pep-treated hearts but not MI + E-pep-treated hearts (Fig. 5e).

### MGF E-domain inhibits cell death in the viable myocardium in vivo

**Fig. 6:**
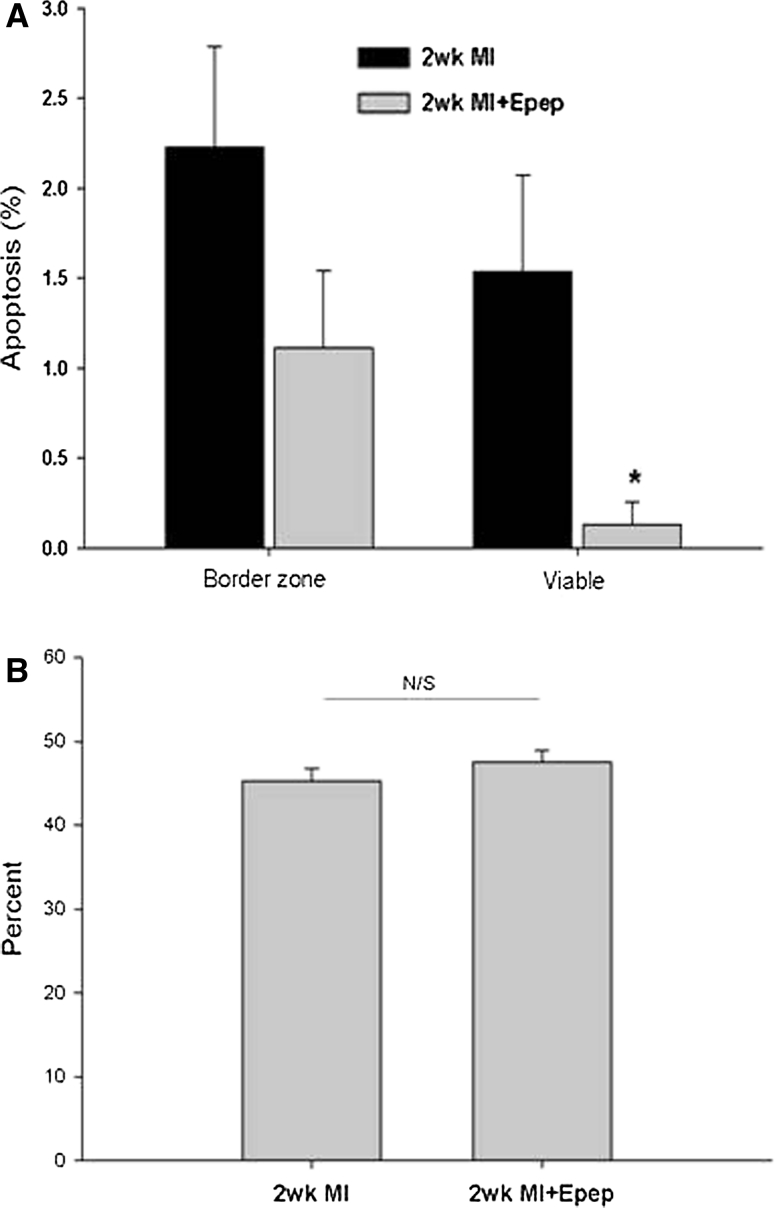
Quantification of cell death in different regions and infarct size in 2 week post-MI mice. **a** The percentage of TUNEL positive nuclei in the border zone and viable myocardium 2 weeks post-MI with E-domain peptide treatment (*P < 0.05 vs. 2 week MI). **b** Infarct size in MI and MI + E-peptide-treated mice (*n* = 5)

The extent of cardiac myocyte cell death was assessed using TUNEL staining in sections cut from MI and MI + E-pep-treated hearts. The numbers of TUNEL positive nuclei were analyzed in the border zone of the infarct and viable myocardium. The data show that while the numbers of apoptotic nuclei tended to be lower in the border zone of MI + E-pep-treated hearts, there was a significant difference in the viable region compared with MI alone (Fig. 6a). Analysis of infarct size showed that infarct size at 2 week was not different between the groups (Fig. 6b). These data suggest that E-peptide treatment may exert a protective effect on the cardiac myocytes in the viable myocardium that would otherwise undergo cell death as a result of the increased mechanical strain following MI.

## Discussion

Myocyte death due to ischemia results in a loss of myocardium, a functional deficit and mechanical overload. In addition, apoptosis also contributes to myocyte drop-out in the remote regions of the myocardium in which there is increased mechanical strain. In order to compensate the remaining cardiac myocytes undergo hypertrophy as a means to normalize wall stress and generate the force required to maintain cardiac output. It has been postulated that apoptosis and hypertrophy that follow mechanical overload are related events, with hypertrophy being initiated by a wave of apoptosis [30]. During these events, local neurohormonal peptides and growth factors such as IGF-1 are produced by the myocardium to mitigate damage. However, these cardioprotective responses are often insufficient to avert the progression of deleterious events but if enhanced could serve as a means of therapeutic intervention. As such, we demonstrate that delivery of a peptide derived from the propeptide form of MGF, an IGF-1 isoform expressed acutely in the heart at time of injury, preserves cardiac function, and prevents cell death and pathologic hypertrophy in post-MI mice. We also demonstrate that the E-domain region prevents apoptosis by inhibiting the intrinsic apoptotic pathway in vitro, correlating this with the anti-apoptotic outcome and preservation of cardiac function with delivery during MI in vivo.

There is strong evidence regarding the importance of IGF-1 as a regulator of myocardial growth and a protector against myocardial cell death in vitro [31, 32]. Also, improvement in cardiac function has been reported following growth hormone administration as a means of increasing systemic IGF-1 levels in animal models [33, 34]. However, these favorable results have not translated well in clinical trials, which do not demonstrate significant prolonged effects of IGF-1 or growth hormone administration [35, 36]. This may not be too surprising since there is scant evidence supporting increased secretion of growth hormone or elevated systemic IGF-1 following MI. In fact, data to the contrary exists showing lower-systemic levels of IGF-1 in patients with acute MI [37]. In addition, the limited bioavailability of IGF-1 once bound to its binding proteins in the circulation is a major therapeutic hurdle. Moreover, these studies speak to the difficulty of defining the effects of IGF-1 produced by the tissue from those of systemic IGF-1 and the assumption that administration of the mature peptide reflects all the biologic actions of IGF-1, when it is produced as a prepropeptide within the physiologic environment.

To address some of these limitations in the context of the heart, cardiac over-expression studies have demonstrated the effects of local IGF-1 and have shown anti-apoptotic actions in myocytes following MI [38]. These salutary effects were attributed to local IGF-1 produced by the muscle even though the liver cDNA used (human class-2 IGF-1Eb) could not be spliced to produce muscle isoforms and resulted in elevated systemic levels of IGF-1 [38]. Likewise, over-expression of the predominant IGF-1Ea isoform also provided protective effects through a combination of anti-inflammatory and anti-apoptotic actions post-MI [39]. However, neither over-expression study addressed the actions of the E-domain independently of the mature peptide. Thus, elucidating which IGF-1 isoform may function in the role of a protector/growth promoter in the heart is important since developing a means to manipulate the IGF-1 system could be advantageous. In this regard, targeting MGF (human IGF-1Ec or rodent IGF-1Eb) as a physiologically relevant isoform is appealing. Data presented here and in other studies corroborate, the MGF isoform is acutely up-regulated with different temporal dynamics than the IGF-1Ea isoform following injury [12, 18]. Furthermore, since the E-domain of MGF appears to be cleaved in injured tissues this raises the possibility that the E-domain may have distinct or synergistic actions to the mature peptide of IGF-1. Supporting this further are an increasing number of studies involving neurological and cardiac models of injury in which delivery of the E-pep of MGF has now been shown to be beneficial [19, 20, 40].

The nature of the molecular mechanism by which the E-domain exerts its effects or influences the actions of IGF-1 still remains to be defined. Our data indicate that a nuclear localization of the endogenous protein and exogenous peptide may be linked to its primary site of action. Also, our data show that the E-pep does not activate the IGF-1 receptor and downstream effector Akt, which form part of the canonical IGF-1 pathway in H9c2 cells. Similar effects have been reported with respect to the E-pep nuclear actions and signaling. Incubation of skeletal muscle myoblasts with an IGF-1 receptor antibody did not block the biological actions of the E-pep in the context of proliferation and migration [17]. Moreover, in neurons and neuronal cell lines subjected to neurotoxic stress, the E-pep did not activate Akt, but selectively increased the expression of heme oxygenase-1 via nuclear localization of Nrf2 which appears to be mediated by PKCepsilon [40, 41]. In H9c2 cells, the MGF E-pep reportedly activates the p44/42 MAPK pathway independently of the canonical IGF-1 pathway [18]. Collectively, these data and ours suggest that the E-pep appears to be able to enter the cell by a yet to be defined mechanism, but results in divergent pathway recruitment that converges on transcriptional regulation as a means of inhibiting cell death.

In addition to the anti-apoptotic effects of IGF-1, the effects on cardiac inotropy and physiological adaptation are well known. In rodent models infusion of both IGF-1 and growth hormone induces cardiac hypertrophy without activation of the fetal gene program and produces a positive inotropic effect [42]. These salutary effects were also demonstrated by using a cardiac-specific IGF-1 over-expression mouse to rescue a dilated phenotype via restoration of calcium dynamics in a model of contractile dysfunction [43]. However, our hemodynamic data do not indicate inotropic actions as a means of explaining the functional preservation observed since control mice treated with the E-peptide did not exhibit increased contractility compared with untreated mice. Rather, our data suggest that the functional response noted in the post-MI mice is likely due to a preservation of contractile function, which could be due to an inhibition of the mitochondrial-mediated cell death pathway of myocytes in the viable myocardium or could arise from preservation of the adult rodent myosin heavy chain isoform (αMHC) expression in those cells. Preventing switching of the αMHC isoform expression to the βMHC often associated with pathologic remodeling could provide a novel means of persevering contractile force. However, within the context of this study in which the peptide was delivered at the time of MI, the causality of this function improvement is difficult to define and would require further studies of delivery over different time points.

The fate of the endogenous E-domain upon cleavage in the cardiac myocyte is a critical issue. Once designated to the secretory pathway, IGF-1 is cleaved by members of the proprotein convertase (PC) family, of which furin is ubiquitous and can act as a cellular endoprotease or extracellular protease. It has been demonstrated that IGF-1 propeptide processing in 293 cells by furin occurs at the Arg^68^–XX–Arg^71^ site between the D- and E-domains to yield the 70 amino acid IGF-1 peptide [44]. However, additional cleavage motifs exist within the E-domain regions of the different isoforms, so development of a stabilized peptide analog was necessary [45]. Consequently, our cellular uptake data indicate that exogenous application of the E-pep utilizes a physiological cell entry mechanism to account for its intracellular actions. The exact mechanism by which this occurs is not known but may involve an interaction with the IGF-1 receptor without activation, a unique receptor-mediated pathway or endocytosis. Whether this reflects the fate and actions of the endogenous MGF E-domain region upon cleavage are still to be determined.

We acknowledge the limitations of this study extend to use of the permanent coronary ligation model, the time of peptide delivery, and the dose and duration of peptide treatment. Nevertheless, our data support the notion that the E-domain regions of IGF-1 isoforms have biological action and provide insight into the function of the MGF E-domain. Furthermore, our data highlight that the regulation of IGF-1 and its actions are more complex than those detailed at the level of the receptor. Consequently, recent interest in defining isoform function has centered on the E-domains since they vary between the different isoforms. Given that tissue stress/injury engages IGF-1 splicing, it is reasonable to postulate that this may underlie the physiologic basis for isoform function. Thus, our data support the concept that modulating IGF-1 isoform function via the E-domain may provide a feasible strategy to lessen overall tissue damage, preserve contractile function, and reduce pathologic remodeling in the heart.

## Electronic supplementary material

Below is the link to the electronic supplementary material.

Supplementary material 1 (DOC 131 kb)
