# Supplementary material for: The E-domain region of mechano-growth factor inhibits cellular apoptosis and preserves cardiac function during myocardial infarction
Source: Mol Cell Biochem. 2013 May 28;381(1):69–83. doi: 10.1007/s11010-013-1689-4 (PMC3720995; doi:10.1007/s11010-013-1689-4)
Supplement: Supplementary file 1 — Supplementary material 1 (DOC 131 kb) [file 11010_2013_1689_MOESM1_ESM.doc]

# Supplementary Data


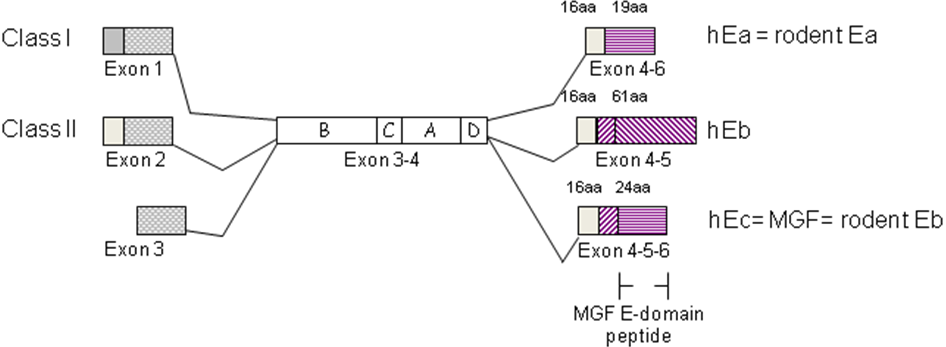


**Figure 1.** Structure of the human *igf-1* gene showing the alternate splicing that produces several isoforms. The 3’-end splicing yields several different prohormone forms with different E-domain regions. The E-domain nomenclature is for human and corresponding rodent isoforms. The 24 amino acid peptide derived for the unique region of the MGF E-domain, (encoded by exons 4, part of 5 and 6), is as shown. The 5’-end splicing yields different preprohormone forms depending on whether exon 1 or 2 are used and are referred to as Class I or Class II transcripts respectively. A cryptic initiation site exists in exon 3 which can also yield a truncated splice variant. (Modified from Shavlakadze T, *et al.,* *Growth Horm IGF Res* 2005; **15**: 4-18.).

**A**


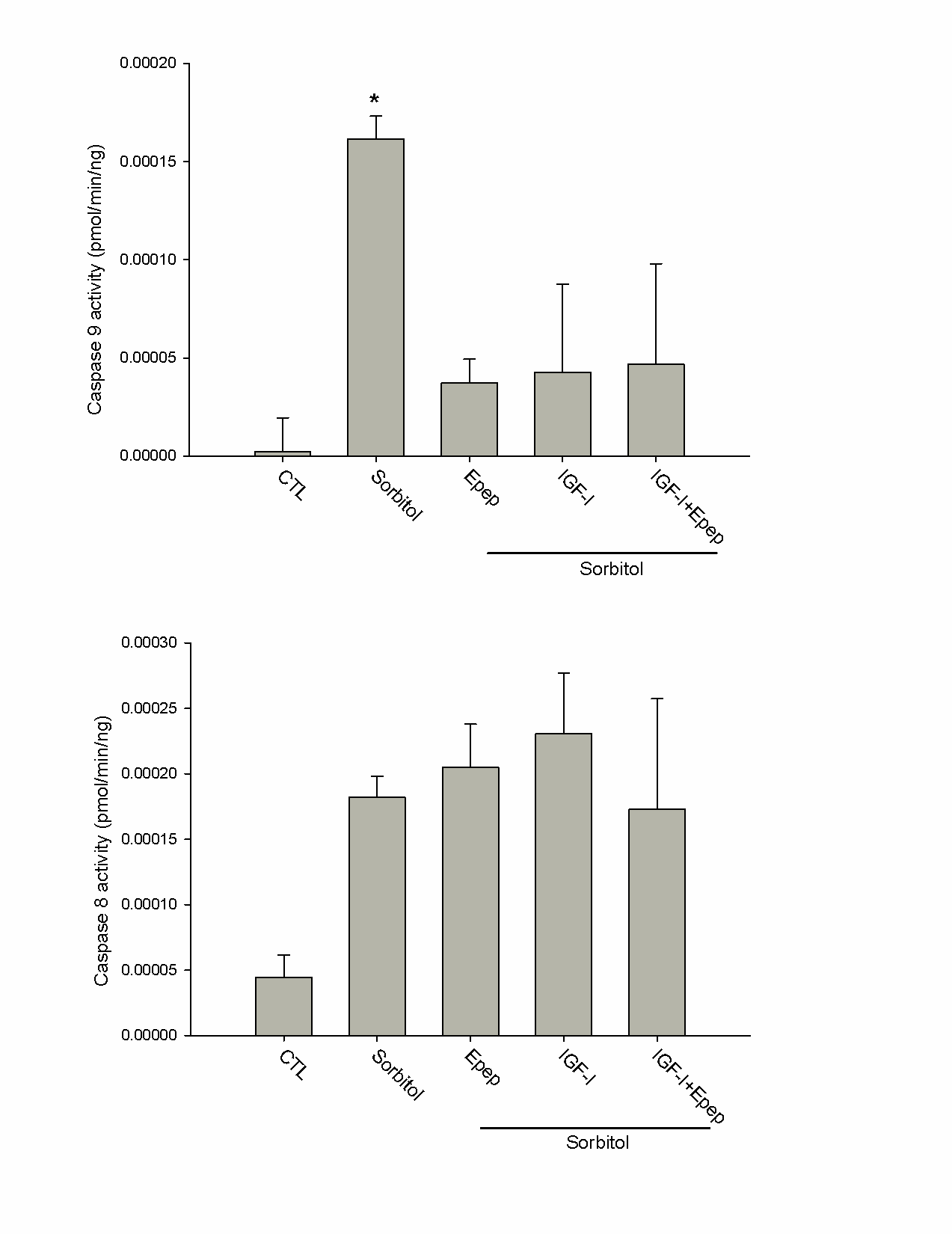

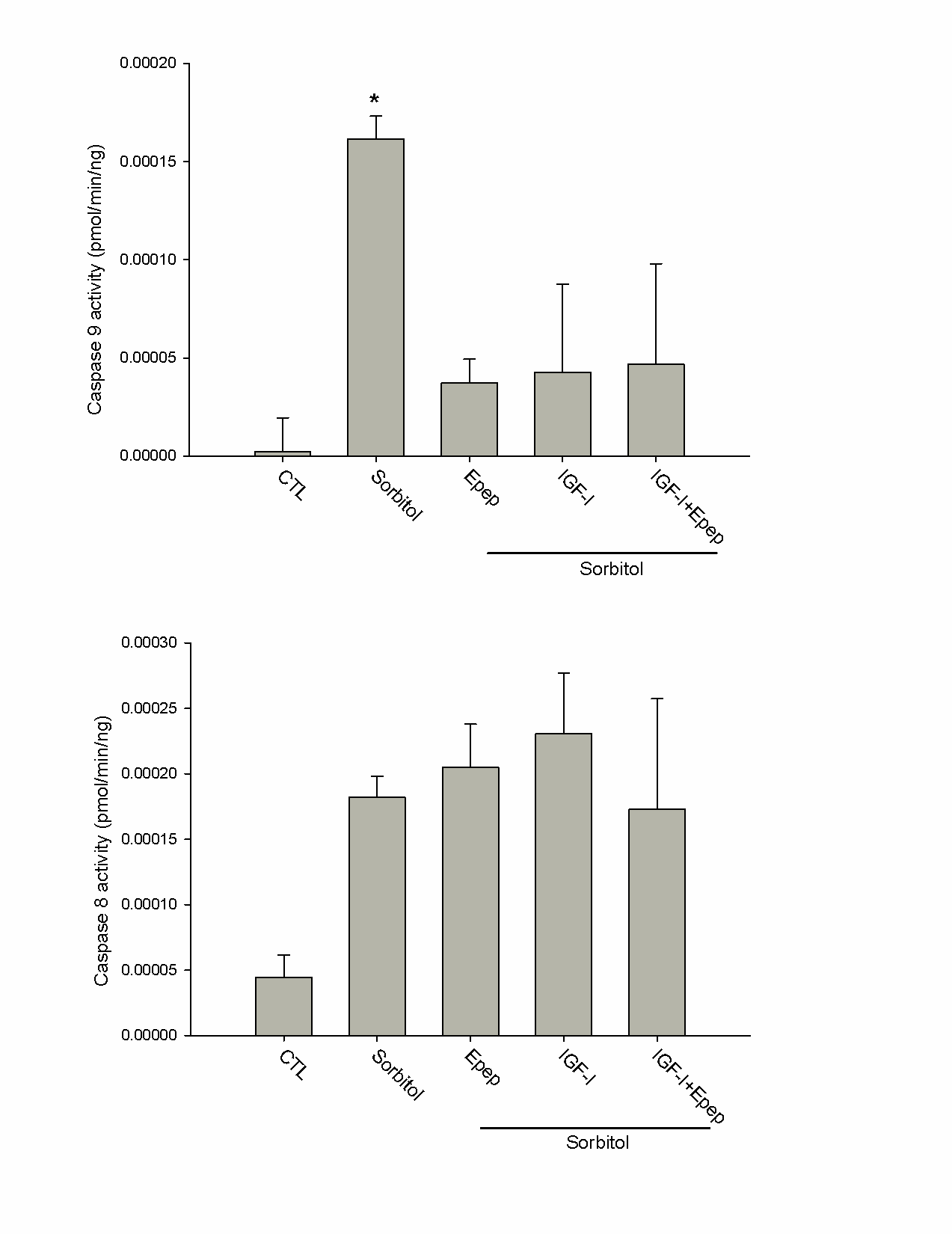


**B**

**Figure 2.** Caspase-9 and 8 activity in response to 0.3M sorbitol treatment (12 hrs), with MGF E-domain peptide treatment (**P*<0.05, *vs.* control, n=3).
